# Using the Global Trigger Tool in surgical and neurosurgical patients: A feasibility study

**DOI:** 10.1371/journal.pone.0272853

**Published:** 2022-08-16

**Authors:** Mareen Brösterhaus, Antje Hammer, Rosalie Gruber, Steffen Kalina, Stefan Grau, Anjali A. Roeth, Hany Ashmawy, Thomas Groß, Marcel Binnebösel, Wolfram Trudo Knoefel, Tanja Manser

**Affiliations:** 1 Institute for Patient Safety, University Hospital Bonn, Bonn, Germany; 2 Central Division Medical Synergies, University Hospital of Cologne, Cologne, Germany; 3 Department of Anesthesiology and Intensive Care Medicine, University Hospital of Cologne, Cologne, Germany; 4 Center of Neurosurgery, University Hospital of Cologne, Cologne, Germany; 5 Department of General, Visceral, and Transplantation Surgery, University Hospital Aachen, Aachen, Germany; 6 Heinrich-Heine-Universität und Universitätsklinikum Düsseldorf, Duesseldorf, Germany; 7 FHNW School of Applied Psychology, University of Applied Sciences and Arts, Northwestern Switzerland, Olten, Switzerland; Public Library of Science, UNITED KINGDOM

## Abstract

**Background:**

The Global Trigger Tool (GTT) has become a worldwide used method for estimating adverse events through a retrospective patient record review. However, little is known about the facilitators and the challenges in the GTT-implementation process. Thus, this study followed two aims: First, to apply a comprehensive set of feasibility criteria to qualitatively and systematically assess the GTT-implementation process in three departments of German university hospitals. Second, to identify the facilitators and the obstacles met in the GTT-implementation process and to derive recommendations for supporting other hospitals in implementing the GTT in clinical practice.

**Methods:**

The study used a qualitative documentary method based on process documentation, with written and verbal feedback from the reviewer, as well as evaluating the study sites during the implementation process. The study was conducted in three departments, each in a different German university hospital. The authors applied a comprehensive set of 22 feasibility criteria assessing the level of challenge in GTT implementation. The results were synthesized and they focused on the facilitators and the challenges.

**Results:**

Of these 22 feasibility criteria, nine were assessed as a low-level challenge, eleven regarded as a moderate-level challenge, and two with a problematic level of challenge. In particular, the lack of time and staff resources, the quality of the information in the patient records, organizational procedures, and local issues, posed major challenges in the implementation process. By contrast, the use of local coordinators and an external expert made important contributions to the GTT implementation.

**Conclusions:**

Considering the facilitators and the obstacles beforehand may help with the implementation of the GTT in routine practice. In particular, early and effective planning can reduce or prevent critical challenges in terms of time, staff resources, and organizational aspects.

## Background

Routinely collected data (including the data in the medical records) was identified as one of the most important components of an effective system for safety measurements that hospitals worldwide should implement [1]. To learn from and act on adverse events (AEs), this was critical to provide suitable instruments for quantifying and characterizing the AEs [2]. The patient record review (PRR) (also known as the medical record review, or the retrospective chart review) is currently the most common method for quantifying patient safety outcomes, such as the AEs, for research purposes [3].

One PRR tool that is used is the Institute for Healthcare Improvement’s (IHI) Global Trigger Tool (GTT). Since its development, the GTT has been used worldwide as a standardized two-stage method for the retrospective PRR of randomly selected patient records [4–10]. International studies have shown that the GTT is an effective tool for an AE assessment in hospitals [11–13]. It has several strengths (for instance, an active involvement of the clinical staff in the quality assurance processes; a standardized and rapid method for recording and monitoring the AEs; and an identification of the AEs, independently of the employees’ willingness to report incidents) [3, 14–16]. The GTT has become a standard measure for patient safety in several countries (for example, Sweden and Norway [17–19]). However, in other countries, such as Germany, the GTT is not yet routinely used for an AE assessment. Furthermore, very limited information is available concerning the feasibility of implementing the GTT into routine practice. A study by Schildmeijer et al. [20] evaluated the process of applying the GTT using focus groups. Adler et al. [21] and Von Plessen et al. [22] both described the lessons learned in setting up the GTT in a hospital setting and they presented recommendations for the implementation thereof, while Adler et al. [21] considered solely the entire implementation process, including leadership commitment, reviewer training, and the review process. Even so, no systematic assessment of feasibility when using predefined criteria to identify the facilitators and the challenges in the implementation process is available. Such information could be used to further promote the GTT implementation internationally.

The authors, therefore, designed this study followed two aims: First, to apply a comprehensive set of feasibility criteria to qualitatively and systematically assess the GTT-implementation process in three departments of German university hospitals. Second, to identify the facilitators and the obstacles in the GTT-implementation process, including local management, reviewer recruitment, reviewer training, patient record review, and the reporting of results to derive recommendations. This would support other hospitals in implementing the GTT, as a patient safety assessment tool in daily clinical practice.

## Methods

### Study object and setting

This feasibility assessment investigated the application of the GTT in a retrospective cross-sectional SafeCulture study. More details of the study are published elsewhere [5]. The study was conducted from 07/2016 to 09/2017 in three departments each in a different university hospital in Germany. The GTT monitors the AEs, by using a series of warning signals, so-called triggers, indicating that an AE might have occurred. In this study, the researchers used two adapted versions of the German GTT, one the PRR in general surgery, and the other in the neurosurgery departments [5]. The general surgery tool included 48 triggers, and the neurosurgery tool had 59 triggers. The GTT was carried out in two departments for general surgery (GS1 and GS2), with 72 and 91 beds, respectively, as well as in one neurosurgery department with 44 beds (NS). The entire implementation process of the GTT is summarized in Table 1. The process involved (1) local management (for example, information and approval from the hospital management and staff council; information on the local staff; and the appointment of a local coordinator); (2) reviewer recruitment; (3) a standardized one-day reviewer training (two-phases), with each reviewer team (consisting of two reviewers and one secondary reviewer) in the three departments; (4) a two-stage PRR process, with primary reviewers (medical students (n = 2), registered nurse (n = 1), physicians (n = 3)), and secondary reviewers (physician (n = 3)), per each hospital. These individuals all possessed the clinical background knowledge about patient record content and the layout, as well as the care provisions [23]; and (5) reporting and discussion of the identified AEs. Before the two-stage PRR, each team was trained on the GTT. The reviewer training was conducted with the support of an external expert. In line with the IHI recommendations, the reviewer training consisted of two phases, without any time limit. In the first phase, two to three local inpatient records per department were reviewed by the primary and secondary reviewers, together with the assistance of an external expert. Afterward, the reviewers discussed their findings and the key findings of each patient record. After the discussion, and with the support of the external expert, the reviewer team agreed on a common understanding of the process and the definitions for PRR. In the second phase, the reviewer team completed a pilot review by using a selected set of three further local inpatient records per department. As well as in the real PRR, and in contrast to the first phase of the training, the primary reviewers reviewed the patient records without the secondary reviewer. The secondary reviewer only assessed the identified adverse events. After the assessment, the found triggers and adverse events were discussed together. The local management and reviewer recruitment began in August 2016, and the reviewer training took place in November 2016 and January 2017. The patient records were drawn from two consecutive months for the patients who were discharged during the given period, between 12/2016 and 01/2017. The two-stage review process was conducted between 01/2017 and 06/2017, depending on the individual time resources of the reviewer within the three departments. The reporting and the discussion of the results took place between 07/2017 and 09/2017.

**Table 1:** Implementation process with the Global Trigger Tool.

**1. Local management**		Organizational information was distributed in the hospitals and the departments; this gained approval from the hospital management and staff council; and the appointment of a local coordinator.
**2. Reviewer recruitment**		Recruitment of the appropriate reviewers.
**3. One-day Reviewer training (two phases)**	First phase	The review of two to three local inpatient records per department by each of the primary reviewers and the secondary reviewer, without time limit.
		Debriefing, with a discussion of the answers and the key points of the review.
		Establishing the rules for the reviewing of the adverse events and noting the determinations of harm.
	Second phase	The review of another three local inpatient records per department by each of the primary reviewers, without a time limit.
		The confirmation of the identified adverse events by the secondary reviewer, who did not review the patient records.
**4. Review process (two-stage)**	First stage	The independent screening of the triggers by two primary reviewers (max. 20 minutes per patient record).
		An in-depth patient record review in the case of an identified trigger, to determine the occurrence of an adverse event.
	Second stage	The confirmation of the identified adverse events by one secondary reviewer.
		The severity categorization of the adverse events, in accordance with the National Coordination Council for Medication Error Reporting and Prevention Index: E = Temporary harm to the patient and required intervention, F = Temporary harm to the patient and required initial or prolonged hospitalization, G = Permanent patient harm, H = Intervention required to sustain life and I = Patient death [24].
**5. Reporting**		The reporting and discussion of the identified adverse events.

### Data collection

The study used a qualitative documentary method [25] to systematically assess the feasibility of the GTT-implementation process. The data for the feasibility assessment was gathered at the Institute of Patient Safety, by two of the research associates, MB and AH, and was supervised by TM, between 07/2016 and 09/2017. For the assessment basis, two different sources were used: 1) Documented process logs on the implementation by the two research associates, MB and AH; and 2) Verbal and written feedback from the reviewers and the local coordinators, by MB maintaining regular telephone and written contact at the three departments throughout the implementation processes (including local management, reviewer recruitment, reviewer training, patient record review, and the reporting of the results).

### Ethical approval

This SafeCulture study was approved by the Ethics Committee of the Faculty of Medicine, the University of Bonn, Germany (Ldf. Nr. 310/14). In addition, this research was sanctioned by the local Ethics Committees of the participating hospitals. Following the data security policies and the German professional code of conduct for physicians (§15 of the professional code), informed consent of the participants was not required, as the data was exclusively collected in-house by the medical staff of the respective hospitals; and the data did not include any personal or identifiable information of the patients, which was respectively analyzed anonymously.

### Data analysis

For the analysis of the process logs, as well as the verbal and written feedback, a set of qualitative feasibility criteria was adapted from Orsmond and Cohn [26]. The adaptation of the set was conducted by the four authors, MB, RG, AH, and TM. In the first step, the three authors, MB, RG, and AH independently rated all of the feasibility criteria from Orsmond and Cohn, concerning their suitability for the study purpose and whether any criteria were missing. The feasibility criteria that were chosen were then compared, discussed, and agreed upon on a consensus involving TM. In a second step, the set of feasibility criteria was adapted for the context of the study and linguistically. For example, Orsmond and Cohn worded the individual feasibility criteria as questions that were allotted into five topics. For better comprehensibility, the authors linguistically adapted the feasibility criteria from Orsmond and Cohn and transformed the questions into statements. For example, criterion 13 was changed from ‘What are the recruitment rates?’ to ‘Sufficient recruitment of appropriate reviewers’. Furthermore, the structure of Orsmond and Cohn’s list of criteria was generally retained but five feasibility criteria were combined from two topics into a new topic. Hence the creation of ‘rating the reviewer recruitment and capabilities’. The feasibility criterion of ‘What are the retention and follow-up rates as the participants move through the study and intervention?’ was deleted. Instead, one criterion to assess the quality of the information in the patient records was added, as this was an important aspect of the implementation process. Moreover, the order of some criteria regarding the implementation process was changed. For the third step, this set of criteria was reviewed by TM, resulting in minor changes, for example, spelling, and the use of terms, prior to finalization.

The final assessment tool consisted of 22 feasibility criteria that concerned five topics: (1) Evaluation of the resources and the ability to manage and implement the GTT (five feasibility criteria); (2) Evaluation of the availability of the patient records and the resulting sample characteristics (six feasibility criteria); (3) Evaluation of the reviewer recruitment and capability (five feasibility criteria); (4) Evaluation and the refinement of the data collection procedures and measures (three feasibility criteria); and (5) Preliminary evaluation of the implementation of the GTT (three feasibility criteria). The adapted and the original feasibility criteria from Orsmond and Cohn are presented in Table 2.

**Table 2:** Adapted and original feasibility criteria from the study and Orsmond and Cohn [26].

Adaption	Original feasibility criteria from Orsmond and Cohn [26]	Adaption (continued)	Original feasibility criteria from Orsmond and Cohn [26] (continued)
Objective 1: Evaluation of the resources and the ability to manage and implement the GTT.	Objective 4: Evaluation of resources and ability to manage and implement the study and intervention	Objective 3: Evaluation of the reviewer recruitment and the capability (These feasibility criteria are a reflection of the feasibility criteria of objective 2).	Combination of objective 1 and 3: Evaluation of recruitment capability and resulting sample characteristics/Evaluation of acceptability and suitability of intervention and study procedures.
Main question: Do the teams in the departments have the resources and the ability to manage the study together with the implementation of the GTT?	Main Question: Does the research team have the resources and ability to manage the study and intervention?	Main Question: Can the departments recruit appropriate reviewers?	Main Question: Can we recruit appropriate participants?/Are study procedures and intervention suitable for and acceptable to participants?
1. Availability of administrative capability, expertise, skills, and time to conduct the study as well as with the implementation of the GTT	1. Does the research team have the administrative capacity, expertise, skills, space and time to conduct the study and intervention?	Not used.	1. What are the retention and follow-up rates as the participants move through the study and intervention?
2. Fulfilment of ethical and data security requirements a) To what extent do the researchers and the reviewers comply with the ethical and data protection requirements for the studies involving the patients?	2. Can we conduct the study procedures and intervention in an ethical manner?	12. Assistance with the recruitment and the organizational obstacles concerning the recruitment a) Are the departments and the local coordinators willing to assist with the recruitment?	4. What are the obstacles to recruitment? (Objective 1)
3. Availability of the technology and equipment to adequately conduct the data collection when using the GTT, including the collection, management, and the analysis of the data a) Are the relevant technologies and equipment available when needed (for example, desk, computer, Excel-template, patient records)? b) What is necessary for the training of the reviewers?	4. Is the technology and equipment sufficient to conduct the study and intervention, including collection, management, and analysis of data?	13. Sufficient recruitment of appropriate reviewers a) How long will it take to recruit enough reviewers? b) What reasons lead to the rejection of the reviewers?	2. What are the recruitment rates? (Objective 1)
4. Implementation of the GTT within the designated budget.	3. Can the study and intervention be conducted within the designated budget?	14. Feasibility and suitability of the inclusion and exclusion criteria for the reviewer selections a) Are the characteristics of the reviewers consistent with the IHI recommendations? b) Are the inclusion and exclusion criteria clear and sufficient, or are they too inclusive or restrictive?	3. How feasible and suitable are eligibility criteria? (Objective 1)
5. Efficient and effective management of the data entry and the analysis with the GTT.	5. Are we able to efficiently and effectively manage data entry and analysis?	15. Feasibility and suitability of the GTT-based data collection for the reviewers a) Does the data collection using the GTT fit with the reviewers’ work activities/work routines? b) Do the reviewers have enough time and capacity for the training and data collection with the GTT? c) To what extent is the implementation of the GTT acceptable for the reviewers?	2. What are the adherence rates to study procedures, intervention attendance, and engagement? (Objective 3)
Objective 2: Evaluation of the availability of the patient records and the resulting sample characteristics.	Objective 1: Evaluation of recruitment capability and resulting sample characteristics	16. Level of the safety procedures for the implementation of the GTT a) Are there any unexpected AEs?	3. What is the level of safety of the procedures in the intervention? (Objective 3)
Main Question: Can the departments select a sufficient sample of patient records?	Main Question: Can we recruit appropriate participants?	Objective 4: Evaluation and refinement of the data collection, procedures, and measures	Objective 2: Evaluation and refinement of data collection procedures and outcome measures
6. Assistance with and the obstacles to the patient record selection a) Are the departments and the local coordinators willing to assist with the recruitment?	4. What are the obstacles to recruitment?	Main Question: How suitable is the data collection when using the GTT for measuring the AEs?	Main Question: How appropriate are the data collection procedures and outcome measures for the intended population and purpose of the study?
7. Availability of potential patients in the respective departments	1. How many potential eligible members of the targeted population are accessible in the local community?	17. Feasibility and suitability of the GTT-based data collection a) New: Have adaptions been made to the GTT and the data collection process? b) Do the reviewers clearly understand the individual triggers and the data collection procedures? c) Do they respond to the triggers with missing or unusable data?	1. How feasible and suitable are the data collection procedures?
8. Is the availability of the patient records in the departments meeting the inclusion and exclusion criteria? a) How many patient records are selected per time unit (two weeks/month)? b) How long does it take to select the patient records? c) How many patient records must be replaced by a replacement record after the inclusion? d) What are the reasons for preventing access to or the use of the individual patient records? What are the reasons for an inability to access or use the individual patient records? e) New: How are incomplete patient records dealt with?	2. What are the recruitment rates?	18. Feasibility and suitability of the amount of data collected with the GTT in general a) Do the reviewers have the capacity to complete the data collection with the GTT as intended? b) Does the overall data collection with the GTT take a reasonable amount of time, or does it create a burden for the reviewers?	2. How feasible and suitable is the amount of data collection?
9. Feasibility and suitability of the inclusion and exclusion criteria for the patient record selection a) Are the inclusion and exclusion criteria clear and sufficient, or are they too inclusive or restrictive? b) Are the characteristics of the patient records consistent with the IHI recommendations?	3. How feasible and suitable are eligibility criteria?	19. Consistency of the data collection when using the GTT a) Were the patient records evaluated consistently in all of the departments?	3. Do the measures appear to be performing in a consistent way with the intended population as compared to measurement information available in the research literature?
10. Dealing with an insufficient quality of the information in the patient records	New developed criterion	Objective 5: Preliminary evaluation for the implementation of the GTT	Objective 5: Preliminary evaluation of participant responses to intervention.
11. Relevance of the implementation of the GTT to the respective departments a) Are there any identifiers in the patient records that suggest the need for an implementation of the GTT?	5. How relevant is the intervention to the intended population?	Main Question: Does the implementation of the GTT show promise of being successful?	Main Question: Does the intervention show promise of being successful with the intended population?
		20. The success of the GTT-implementation, as shown in the collected data a. Do the results of the GTT suggest that the implementation of the GTT has promise?	1. Does examination of quantitative data suggest that the intervention is likely to be successful?
		21. The success of the GTT-implementation, as shown by the feedback of the people involved in the GTT-implementation.	2. Do participants or relevant others provide qualitative feedback that may be indicative of the likelihood that the intervention will be successful?
		22. Indications for the failure of the GTT-implementation in general. a) Is there any evidence that the GTT was not implemented in an intended manner? b) Will there be further adaptations of the data collection process when using the GTT to better evaluate the data from the patient records? c) New: Over what period of time is the data collection carried out when using the GTT? d) Are the data collection procedures and the outcome measures suitable for the study? e) Are the results consistent with the results of previous studies?	3. If the quantitative and/or qualitative data suggest that the intervention is not promising

AE, adverse events; GTT, Global Trigger Tool

Based on the documentation and guided by the feasibility criteria, MB, RG, and AH synthesized the process logs and the verbal and written feedback, focusing on the facilitators and the challenges in the implementation process, by allocating them to the individual feasibility criteria. MB and AH then independently assessed the level of challenge during the implementation process for each criterion. Again, by involving TM in the next step, the results of the independent assessment were combined, compared, discussed, and agreed on a consensus between the three authors. For a better understanding, the single feasibility criterion was visualized as follows: red = problematic (several challenges), yellow = moderate (a few challenges), and green = low (almost no challenges). Based on this synthesis, the authors critically discussed and derived general recommendations regarding the five topics to support the hospitals in their approach when implementing the GTT into local practice.

## Results

Out of the 22 feasibility criteria, nine were assessed with a low level of challenge, eleven with a moderate challenge, and two with a problematic level of challenge. Table 3 provides an overview of the levels of challenge during the GTT implementation for all of the criteria. The following syntheses of the results are structured according to the five topics and the 22 feasibility criteria from Orsmond and Cohn [26].

**Table 3:** The level of challenge is identified by the feasibility criteria for the implementation of the GTT.

Feasibility criterion	Level of challenge 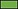 low 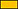 moderate 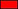 problematic
**Resources and the ability to manage and implement the GTT.**	
1. Availability of administrative capability, expertise, skills, time to conduct the study as well as with the implementation of the GTT.	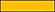
2. Fulfilment of ethical and data security requirements.	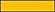
3. Availability of the technology and the equipment to adequately conduct the data collection when using the GTT, including the collection, management, and the analyses of the data.	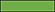
4. Implementation of the GTT within the designated budget.	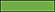
5. Efficient and effective management of the data entry and the analysis with the GTT.	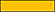
**Availability of the patient records and the resulting sample characteristics**	
6. Assistance with and the obstacles to the patient record selection.	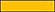
7. Availability of potential patients in the respective departments.	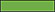
8. Availability of the patient records in the departments meeting the inclusion and exclusion criteria.	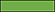
9. Feasibility and suitability of the inclusion and exclusion criteria for the patient record selection.	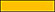
10. Dealing with an insufficient quality of information in the patient records.	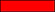
11. Relevance of the implementation of the GTT to the respective departments.	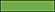
**Reviewer recruitment and capability**	
12. Assistance with the recruitment and the organizational obstacles conerning the recruitment.	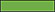
13. Sufficient recruitment of appropriate reviewers.	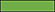
14. Feasibility and suitability of the inclusion and exclusion criteria for the reviewer selection.	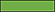
15. Feasibility and suitability of the GTT-based data collection for the reviewers.	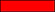
16. The level the safety procedures for the implementation of the GTT.	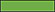
**Data collection procedures and measures**	
17. Feasibility and suitability of the GTT-based data collection.	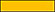
18. Feasibility and suitability of the amount of data collected when using the GTT in general.	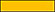
19. Consistency of the data collection when using the GTT.	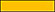
**Implementation of the GTT**	
20. Success of the GTT-implementation, as shown in the collected data.	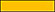
21. Success of the GTT-implementation, as shown by the feedback of the people involved in the GTT-implementation.	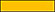
22. Indications for the failure of the GTT-implementation in general.	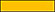

GTT, Global Trigger Tool

### Resources and the ability to manage and implement the GTT

Out of the five feasibility criteria in this topic area, two were assessed as presenting minor challenges, and three were designated as moderate challenges.

#### (1) Availability of administrative capability, expertise, skills, time to conduct the study, and the implementation of the GTT (moderate level of challenge)

The implementation of the GTT was supported by a local coordinator that was experienced with the local requirements, and this person organized the implementation process at each site (for example, reviewer recruitment, selection of the patient records, organization of the equipment for the data collection). Prior to the PRR, the reviewer training was conducted with an external expert, who was familiar with the GTT application but who was not directly involved in the local practices. The external expert was important for ensuring that the local reviewers understood the purpose of the measurements. Additionally, the external expert maintained a neutral point of view in the initial reviews, while providing advice, as appropriate. However, while the external expert was planned and budgeted in the study, the involvement of an external expert would require additional financial resources and efforts in engagement by the hospitals. Concerning the general management of the implementation of the GTT, the process was properly scheduled, by ensuring that there was enough time for training the reviewers in each department, and for preparing and conducting the data collection when using the GTT.

#### (2) Fulfillment of ethical and data security requirements (moderate level of challenge)

For the entire study, approval was obtained from the Ethics Committee of the Medical Faculty of the University of Bonn, Germany. As the implementation of the GTT was part of a research project, all of the physicians involved in the data collection required ethical and professional counseling, in accordance with German law (Art. 15 of the Professional Code for Physicians in Germany). Moreover, clarification on data security and ethical issues for all members of the reviewer teams was ensured. A minimal challenge in direct communication between the physicians and the responsible ethical committees was experienced. The entire process of ethical and professional counseling took, on average, three to four weeks. In Germany, only professionals who work in the departments treating the patients have access to the patient records. Thus, the main challenge in this area was the lack of access rights for the reviewer teams to the patient records in GS1 and NS at the beginning of the data collection, as the reviewers did not work in these departments. Furthermore, the physicians had more extensive access rights to the patient records than the other professionals, such as the medical students. Therefore, the first step was to set up full access rights to the patient records. In GS2, no problems occurred, as all of the reviewers were physicians and they had full access to the patient data.

#### (3) Availability of the technology and the equipment to adequately conduct the data collection using the GTT, including the collection, management, and the analyses of the data (low level of challenge)

The organization of the training equipment (GTT manual, computer with access to the patient records, GTT-template in Excel, and a total of five to six patient records per department for the training) was a minor challenge. For the data collection, the reviewer required a workspace equipped with a computer, with access to the patient records, and the GTT template in Excel. Concerning the workspace, GS1 conducted the PRR in a meeting room that was often occupied, despite being reserved, so another room had to be found at short notice.

#### (4) Implementation of the GTT within the designated budget (low level of challenge)

The implementation of the GTT did not require extensive financial resources for the data collection equipment. An external expert for the reviewer training, as well as the reviewer’s reimbursement to cover expenses, was previously budgeted. Thus, the implementation of the GTT was within budget. However, while all the three hospital managements supported the implementation of the study, none of them could provide any additional resources to exempt the reviewers from their daily work during the PRR.

#### (5) Efficient and effective management of the data entry and analyses when using the GTT (moderate level of challenge)

The data was collected by primary reviewers in the departments at the scheduled times. However, the need for scheduling appointments with a secondary reviewer for confirming and categorizing the severity of the AEs posed a moderate challenge for the reviewers.

### Availability of the patient records and the resulting sample characteristics

The availability of the patient records and the resulting sample characteristics were categorized as a low level of challenge in three out of the six feasibility criteria. Two of the feasibility criteria resulted in moderate challenges and one was a major challenge.

#### (6) Assistance with and the obstacles to the patient record selection (moderate level of challenge)

The patient record selection was carried out by the local coordinators. In GS1, the coordinator was supported by a primary reviewer, and in GS2 and NS, by the respective department office. For the randomized patient record selection, the patients were filtered by the discharge date. The initial difficulties with the hospital information system in GS1 prevented the filtering regarding the discharge date but these problems were solved with the support of the departmental staff.

#### (7) Availability of potential patients in the respective departments (low level of challenge)

Each department had a sufficient patient volume for a random selection of 40 patient records (20 per month over a period of two consecutive months, by those patients that were admitted between 01/2017 and 03/2017).

#### (8) Availability of the patient records in the departments that meet the inclusion and exclusion criteria (low level of challenge)

In each department, all of the randomly selected patients met the IHI recommendations for the inclusion criteria (patient stay > 24h, ≥18 years, discharged for at least 30 days, with a complete patient record). This allowed for an oversampling of 12 additional patient records (six per month and the department named) for any replacement required. However, only one patient record had to be substituted in GS1 since the original patient record was assigned to another department during the period of the PRR, thus it was inaccessible.

#### (9) Feasibility and suitability of the inclusion and exclusion criteria for the patient record selection (moderate level of challenge)

In all of the departments, the inclusion criterion of a "complete patient record" only became apparent during the PRR, as some documents, such as extra medication plans, only became available in-patient records, weeks after the discharge date. In these cases, the PRR was interrupted and the patient records were marked and reviewed after the documents were added.

#### (10) Dealing with an insufficient quality of the information in the patient records (problematic level of challenge)

All of the departments were in transition from a solely paper-based documentation to electronic patient records. As part of this process, the patient records were scanned after the patient’s discharge and then accessed as digital patient records in the local clinical information systems. While the quality of the scanned documents was generally good, it was detrimental when red ink, pencil, and colored templates were sometimes not scanned appropriately and thus not visible. Likewise, the handwriting on the scanned documents was often illegible. Accordingly, some triggers could not be evaluated appropriately. In accordance with the IHI, the GTT trigger was only rated if the reviewer identified relevant information in the PR. The reviewers were instructed to assign triggers wherever the information was not applicable, or where it had not been documented, with an ‘NA’. While the risk of overlooking the information was covered by the independent review process in the first stage, there was no information on the completeness and the quality of the patient’s record contents.

#### (11) Relevance of the implementation of the GTT to the respective departments (low level of challenge)

All of the departments identified between ten and twenty-four AEs [5], with most of the AEs identified as temporary harms requiring an intervention (category E), or initial or prolonged hospitalization (category F). The local coordinator gave feedback that to their knowledge, the harms categorized in E and F had not been revealed by other methods (administrative routine data, critical incident reporting). In GS2 and NS, the AEs of severity categories E and F (for example, intraoperative blood loss, pneumonia) occurred repeatedly in the reviewed patient records.

### Reviewer recruitment and capability

The reviewer recruitment and capabilities were associated with five feasibility criteria, of which four were rated with a low level of challenge, and one with a problematic level of challenge.

#### (12) Assistance with the recruitment and the organizational obstacles concerning the recruitment (low level of challenge)

The participating hospitals’ management and staff council was informed about the procedures and approval was received, with full support prior to implementing the GTT. Moreover, the reviewer recruitment was conducted and organized by a responsible local coordinator, who provided all of the required information to the potential reviewers and served as a contact in case of queries.

#### (13) Sufficient recruitment of appropriate reviewers (low level of challenge)

The effort and time required for the recruitment varied across the departments, due to differing staff resources. However, each department was able to recruit a sufficient number of appropriate reviewers within the scheduled time.

#### (14) Feasibility and suitability of the inclusion and exclusion criteria for the reviewer selection (low level of challenge)

As recommended by the IHI, all of the departments designated at least two primary reviewers and one secondary reviewer, with all of the secondary reviewers being physicians. However, due to staff resources, the composition of the reviewer teams varied across the departments. In all of the departments, the IHI recommendations were followed in the recruitment of the reviewers (reviewer team with a clinical background, knowledge of the patient’s record content and layout, as well as care provision at the hospital) [23]. In NS, only the secondary reviewer worked in the department, while the two primary reviewers were a physician and a registered nurse working in the hospital’s quality management department. In GS2, the reviewer team consisted of three physicians working in the department. In GS1, the reviewer team was solely supported by one local physician, who served as a secondary reviewer. Since the GTT was used for the first time within a SafeCulture study in Germany, the primary and secondary reviewers of the three departments had no expertise in PRR with the GTT. Due to a lack of personnel, two medical students served as primary reviewers, thus they required additional support and training to become familiar with the patient records. Overall, and despite the different compositions of the reviewer teams, the IHI recommendations were met, and all of the teams successfully completed the PRR.

#### (15) Feasibility and suitability of the GTT-based data collection for the reviewers (problematic level of challenge)

In all of the three departments, the reviewers reported that the PRR was not exclusively carried out during the reviewers’ regular working hours but it required overtime. Due to workload and the composition of the reviewer teams, especially in NS and GS1 (such as physicians from the departments versus the medical students and personnel from the other departments), the primary and secondary reviewers had difficulties scheduling the discussion of the results in the second stage of the PRR process. Consequently, the categorization of the severity of the identified AEs was delayed in these two departments.

#### (16) Level of safety of the procedures for the implementation of the GTT (low level of challenge)

While the participating hospitals did not exempt the reviewers from their daily work, for PRR, the data collection was conducted on top of their regular clinical duties. This additional workload, with working overtime, may pose a risk to patient care. However, within this study, no additional safety issues other than those that were measured with the GTT were identified.

### Data collection procedures and measures

In this section, all of the three feasibility criteria were classified as a moderate level of challenge.

#### (17) Feasibility and suitability of the GTT-based data collection (moderate level of challenge)

Before the data collection, the authors used the German version of the GTT [27], and for the neurosurgery department, selected triggers from the National Institute for Health and Welfare of Finland were used [28]. Both of them were slightly adapted in terms of linguistics and content. New triggers were only added when the essential required content was not covered by existing triggers (for instance, ‘relevant increase of leukocytes or other serological infection values during the hospital stay’). To ensure consistent data content, no triggers were removed from the GTT. However, the GTT required some local tailoring to the different conditions in the individual departments (for example, the different use of drug names). In addition, the order of the triggers was changed in some departments, to reflect the composition of the local patient records, and to facilitate continuous reviewing, without jumping between the triggers or the patient record documents. To facilitate the trigger search and the evaluation, and to ensure the reviewers understood the individual GTT triggers, the Excel template for the data entry contained additional information on each trigger (for instance, specific examples of the adverse events) and guidelines for completing the form. Hence, the missing data resulted only from the missing or unidentifiable information in the patient records.

#### (18) Feasibility and suitability of the amount of data collected when using the GTT in general (moderate level of challenge)

In GS2, the secondary reviewer verified the AEs found and determined the severity immediately after the primary reviewers screened the patient records. In contrast, the PRR in GS1 and NS delayed the data collection, due to additional appointments that were required for discussions between the primary and the secondary reviewers. For the GTT, the average PRR time was limited to 20 minutes per patient record. To ensure compliance with the recommended 20 minutes per patient record, stopwatches were used in all departments. The reviewers reported that this was particularly helpful for their own control, as there was often a risk that they would start reading the patient records in their entirety, instead of screening them. Still, the local coordinator informed that the average time for the PRR varied, and in some cases, the reviewers required more time. This especially became prevalent in the early stages of the PRR, or in the extensive patient records. Nonetheless, all of the reviewers improved rapidly, as they practiced their reviewing skills and became more familiar with the local documentation standards and patient record structures. This became more apparent when the PRRs were performed by students or quality managers, who were less familiar with the local documentation standards in the patient records. Based on their limited experience, the students had more difficulty evaluating the medical triggers than did the physicians or nurses.

#### (19) Consistency of the data collection when using the GTT (moderate level of challenge)

In order to ensure consistent data collection, a one-day reviewer training was conducted, providing all of the reviewers with uniform definitions and an explanation of the triggers. The AE assessment and the categorization of AE severity were in accordance with the National Coordination Council for Medication Error Reporting and Prevention Index [24]. During the training and the data collection, the professionals often struggled with the categorization of the AEs. This typically happened with the harms of severity, namely, the categories E and F, with temporary harm to patients requiring an additional intervention, or an initial or prolonged stay, respectively. During the training, the professionals tended to downplay the identified AEs. This was not because they considered them unimportant. This was because the GTT does not distinguish between avoidable and unavoidable AEs, and minor interventions were considered an important treatment to the patient, but not harmful. For example, before the training, the professionals understood the use of restraints (one trigger of the general module of the GTT), as a measure to strap patients, as a means to protect them from injuries. However, this trigger may either indicate that the AEs were caused by the restraint (bruises or anxieties), or the restraint was a consequence of an AE (delirium). By using the GTT, this trigger would identify an AE in both cases, even if greater harm (a fall) could have been prevented through this intervention. If required, the research team was consulted during the data collection to answer questions on the categorization of patient harms, as this was complex and highly dependent on professional experience.

### Implementation of the GTT

Overall, the implementation of the GTT resulted in three feasibility criteria, with a moderate level of challenge.

#### (20) Success of the GTT implementation, as shown in the collected data (moderate level of challenge)

The identification of 53 AEs proved that the GTT was successfully implemented, supporting the importance of a GTT implementation. This was supported by the statement of the reviewers, that most of these AEs, especially those categorized in E and F, would have not been identified through other measures (administrative routine data, critical incident reporting). This was also reflected by the reviewer feedback that was in favor of the GTT and its further use. Some efforts were made at GS1 and NS to continue the use of GTT. For example, the implementation was continued in GS1 as part of a doctorate thesis. The hospital of the NS department adapted the tool for perinatology.

#### (21) Success of the GTT implementation, as shown by the feedback of the people involved in the GTT implementation (moderate level of challenge)

The reviewers found the results very valuable. However, it was also noted that staff resources and performing the PRR outside of working hours present an obstacle. During the PRR, no changes were made to the instrument. However, the reviewers made some suggestions for improving the GTT template and some triggers (for example, adding a row in the template for special information per patient record and the adjustment of triggers based on specific departmental conditions).

#### (22) Indications for the failure of the GTT implementation in general (moderate level of challenge)

Despite some deviations from the IHI recommendations (for example, the composition of the reviewers), the GTT was generally implemented in the intended manner. Further adaptations to the tool were not required during the data collection. The identification of the AEs revealed four recurring AEs in two departments. However, prolonged use of the GTT independently from this study was recommended and both the local coordinators and the reviewers emphasized the importance of the GTT. An integration ideally into routine patient safety activities within the departments was encouraged; however, none of the participating departments was able to invest further resources for continuing the GTT reviews.

### Facilitators and the obstacles in the GTT implementation process

As a result of the systematic assessment regarding the feasibility of the GTT implementation, a list of recommendations regarding the facilitators and the obstacles in the GTT-implementation process was summarized (Table 4). This list was structured along with the five feasibility topics, as it was designed to support the hospitals in the implementation process of the GTT when rendered in daily clinical practice.

**Table 4:** Recommendations regarding the facilitators (F) and the obstacles (O) in the GTT-implementation process.

Resources and the ability to manage and implement the GTT
F: Ensure active support of the hospital management and the professionals involved at the department level.
F: Appoint a responsible coordinator in the implementation process, who supports the general administration processes (for example, reviewer recruitment) and serves as a contact person for the reviewers.
F: Consider the ethical aspects of the data protection and schedule sufficient time to gain approval of the ethics and staff council, if required.
F: Schedule sufficient time for reviewer training in each department.
F: Involve an external expert for the initial training and the repeated training during the data collection.
F: Take care of organizational obstacles (access rights and the required equipment).
O: Lack of time arranging the appointment between the primary and the secondary reviewer, as this may cause a time delay.
**Availability of the patient records and the resulting sample characteristics**
F: Test the programs for the randomized selection of the patient records (especially concerning the filtering by the patient discharge date).
F: If the scan quality of the documents is very poor, organize the original documents for the PRR.
O: Incomplete records pose a risk of biased/falsified results.
**Reviewer recruitment and capability**
F: Schedule enough time for the reviewer recruitment and involve the department and external staff in the recruitment process.
F: Make sure that the medical students have sufficient medical knowledge and clinical experience.
F: Ensure that all of the reviewer teams are at the same level of knowledge.
O: No exemption of the reviewers to conduct the PRR from their regular responsibilities.
**Data collection measures and procedures**
F: Provide the reviewers with additional information (examples of the AEs) and instructions for filling out the GTT, to facilitate the trigger search and analysis.
F: Use a stopwatch for keeping an average of 20 minutes per patient record.
F: Provide a uniform definition of the AEs and check the reviewers’ assessments based on the definition throughout the PRR.
F: Ensure very close contact between the local coordinator and the reviewers throughout the review process to monitor the reviewers’ methods and attitudes, and respond to questions so that input errors and comprehension problems can also be avoided.
**Implementation of the GTT**
F: Use the GTT continuously for obtaining a representative picture of the situation in the department and for noticing any changes.
O: Use the GTT as an instrument for solely comparing the AE rates.
O: Notice that for the continuous use of the GTT, further resources have to be invested.

AE, adverse events; GTT, Global Trigger Tool; PRR, patient record review

## Discussion

This is the first study, which has systematically assessed the feasibility of the GTT implementation process (including local management, reviewer recruitment, reviewer training, patient record review, and the reporting of the results) when using 22 feasibility criteria and derived recommendations in the three hospitals. The study has revealed various levels of challenge. The criteria with a problematic level of challenge (two out of 22) mainly involved either a poor quality of the information in the patient records or a lack of time, especially due to the time-consuming activities (for example, the meetings with the secondary reviewer). The challenges regarding the quality of the patient records have also been described in other studies. In particular, insufficient documentation was noted on several occasions. For example, the AEs were not documented, and accordingly, they could not be identified in the patient records, due to different standards, clinical ignorance, and worries about the liability risks [17, 20, 29]. This was because scanning the original patient records to create an electronic patient record affected the quality of readability [30], with the use of the original patient records, instead of illegible scans, is recommended. Concerning the time resources, it is also recommended to consider alternate reviewer compositions (for example, involving the medical students) and ensure that the reviewers are relieved of some of their daily tasks. Even this is in discrepancy with Adler et al. [21], who solely recommended reviewers with at least five years of experience in the clinical field, and even students with enough experienced medical knowledge, with at least some clinical experience, who could then be eligible for the PRR.

The criteria that were classified as a moderate level of challenge (eleven out of 22), mostly involved the organizational procedures and the local issues (for example, planning required for the local tailoring of the GTT and the data collection procedure), or a lack of staff resources (for instance, the inability to exempt the reviewers from their daily tasks). Moreover, this moderate challenge also presented the need for an external expert. This expert should be familiar with the GTT and, even more importantly, not be directly involved in the local practices. The external expert would be an important facilitator, ensuring a neutral point of view in the reviewer training and the initial PRR, making objective remarks and recommendations regarding the data collection and evaluation. This became most obvious during the training when the professionals tended to downplay the identified AEs. Through the external expert, the study was able to inform reviewers of the importance of their decisions. This was especially important during the sampling procedure and the data collection process, as an expert can oversee the PRR and counteract the sampling biases [22]. Consequently, repetitive trainings and discussion of the identified AEs, within the reviewer teams, would ensure a shared understanding and a consistent application of the GTT [31]. Thus, the hospitals should, therefore, train several reviewers simultaneously as a team [22].

Prior to the data collection, it was important to adapt the GTT to the local conditions in the hospitals. Since the tool does not cover all specialties and department-specific situations, there is a conflict between when using the tool and adapting it locally, which can result in significant deviations from the GTT. With the development of triggers and new modules, clinical relevance and benefit must be considered and continuously evaluated [32]. Another facilitator was the supplementary information provider on each trigger and guidelines for completing the form, which simplified the trigger search and the evaluation. The moderate level of challenge additionally resulted from the time limit of 20 minutes per patient record, and this was difficult to be administered. This challenge was also found by Schildmeijer et al. [20]. Therefore, stopwatches are recommended. Further to identify the recurring AEs and allow more time for the detailed analyses, the hospitals must use the GTT for a longer period of time than was possible in this study. Only then would it be possible to investigate if the AEs were unique events or a consequence of problematic structures and practices in the department (routine use of restraints instead of choosing alternatives). Moreover, using additional data (administrative routine data, or critical incident reporting) and more detailed analyses, such as root-cause-analyses, can support the identification of problematic structures and processes, and thus learn from the errors and improve the patient safety work [33, 34]. Furthermore, when using and analyzing the GTT, different additional data should be noted and critically reviewed to learn for improvement. For example, Hibbert et al. 2016 [35] recommended that the AEs that were associated with the omissions and the preventability scores for a priority setting were important data, which should be considered. The focus of using the GTT should not be exclusively on counting the AEs. Rather, the aim of use should also be able to understand and characterize the AEs.

The lowest level of challenge (nine out of 22 feasibility criteria) was identified in terms of support by the hospital management and the professionals in the departments through the GTT-implementation process.

Moreover, the designation of at least one responsible person (for instance, the local coordinator), who is acquainted with the local processes and coordinates the reviewer recruitment, together with the organization of the required equipment (for example, for the training and the data collection), was proved to be an important facilitator in the implementation process.

### Limitations of the study

For assessing the feasibility of the GTT implementation, the feasibility criteria that were originally developed were adapted to assess the feasibility of the interventional studies. The evaluation of the GTT implementation was limited to a two-month data collection, within the three departments. Thus, the challenges that were identified were not necessarily generalizable but they have provided a realistic picture of the challenges in the GTT-implementation process. Besides, these results have related to the implementation of the GTT in Germany. Some of the obstacles arose exclusively from specific circumstances (health care system/data protection) in Germany. As a result, some of the recommendations regarding the facilitators might be specific to Germany. Nonetheless, it is believed that most of the recommendations apply to other countries as well, especially those that have not yet implemented the GTT as a routine assessment. Furthermore, this GTT implementation was conducted in the framework of a study, which comes with the support of the researchers. Facilitators and challenges may arise in any setting, without such support. However, the results of this study can provide initial insights into the feasibility of a GTT implementation. In the context of the SafeCulture study, the review process was restricted to two months. Be that as it may, the implementation of the GTT requires local management, reviewer recruitment, reviewer training, and the reporting of the results, together with the effort required regarding these parts, in a setting without a study context. Lastly, but not least, the results of this study might have been biased by the researcher’s personal experiences and assumptions prior to the study. These have been reduced to a minimum by the independent ratings in the selection of feasibility criteria for the assessment (rating of criteria) itself, by MB, RG, and AH. Moreover, all of the results and decisions have been discussed and agreed on a consensus when involving TM.

## Conclusion

The GTT implementation in practice comes along with various levels of challenge. Thus, the success of a GTT implementation process might be supported by considering potential facilitators and obstacles before the implementation process begins. Through a systematic assessment of the feasibility, essential aspects to be considered during the GTT-implementation process were derived. In particular, critical challenges relating to time, staff resources, and organizational aspects can be reduced in advance through effective planning. The identification of the AEs, especially the severity categories E and F, illustrates the GTT relevance for the departments. While the harms of categories G and I might be identified through other methods (for example, administrative routine data, and critical incident reporting), the harms of categories E and F have not been exposed in this study. By identifying these AEs with the GTT, problematic processes and structures can be identified early, and more serious harms may be avoided. The GTT can thus be a valuable supplement to other instruments for measuring the AEs.
